# Protective Effect of Ethyl Rosmarinate against Ulcerative Colitis in Mice Based on Untargeted Metabolomics

**DOI:** 10.3390/ijms23031256

**Published:** 2022-01-23

**Authors:** Baisong Zhou, Juntong Liu, Yaru Wang, Fulin Wu, Caixia Wang, Cuizhu Wang, Jinping Liu, Pingya Li

**Affiliations:** 1 School of Pharmaceutical Sciences, Jilin University, Changchun 130021, China; bszhou19@mails.jlu.edu.cn (B.Z.); jtliu20@mails.jlu.edu.cn (J.L.); flwu20@mails.jlu.edu.cn (F.W.); cxwang20@mails.jlu.edu.cn (C.W.); wangcuizhu@jlu.edu.cn (C.W.); 2 College of Basic Medical Sciences, Jilin University, Changchun 130021, China; yaru20@mails.jlu.edu.cn; 3 Research Center of Natural Drug, Jilin University, Changchun 130021, China

**Keywords:** ulcerative colitis, ethyl rosmarinate, anti-inflammatory activity, metabolomics

## Abstract

Aiming at assessing the therapeutic effect of ethyl rosmarinate (ER) on ulcerative colitis (UC), the following activities were performed in vitro and in vivo in the present study. Firstly, a lipopolysaccharide (LPS)-induced RAW264.7 cell inflammation model was established to determine the level of inflammatory factors. Then, a UC mice model induced by dextran sodium sulfate (DSS) was established to further investigate the effects of ER on symptoms, inflammatory factors and colon histopathology. Finally, serum and colon metabolomics studies were performed to identify the biomarkers and metabolisms closely related to the protective effect of ER on UC. The results showed that after ER intervention, the levels of inflammatory factors (NO, TNF-α, IL-1β and IL-6) and key enzyme (MPO) in cell supernatant, serum or colon were significantly decreased, and the disease activity index and colon tissue damage in mice were also effectively improved or restored. In addition, 28 biomarkers and 6 metabolisms were found to be re-regulated by ER in the UC model mice. Therefore, it could be concluded that ER could effectively ameliorate the progression of UC and could be used as a new natural agent for the treatment of UC.

## 1. Introduction

Ulcerative colitis (UC), a globally prevalent form of inflammatory bowel disease, often causes bloody diarrhea, weight loss, rectal bleeding, and even significantly increases the risk of colorectal cancer [1,2,3]. It has been proven that UC was mainly affected by inflammation, colonic barriers, the immune system, microbiota, genetics and environmental factors [4,5,6]. Currently, drug intervention is the main method for the treatment of UC. The drugs commonly used in clinics are 5-aminosalicylic acid, sulfasalazine, dexamethasone and infliximab, etc. However, there are still some disadvantages such as the unsatisfactory curative effect, poor oral absorption, low tolerability and high cost. It is necessary to develop novel anti-UC drugs. Natural medicine, with unique efficacy, multiple targets, toleration, biocompatibility, low toxicity and a realistic price advantage is receiving more and more attention [7].

Rosmarinic acid, a natural product existing in many plants [8,9,10], was regarded as a potential agent for ameliorating colitis and preventing colon cancer [11,12]. However, the low bioavailability caused by the inefficient transmembrane absorption weakened the biological activity of rosmarinic acid [13]. Interestingly, compared with rosmarinic acid, ethyl rosmarinate (ER, CAS: 174591-47-0) showed better anti-inflammatory activity [14]. Besides, ER was also reported to have other activities such as anti-oxidation [15], anti-hypertension [16] and neuroprotective activity [17]. For example, ER showed an ability to inhibit LPS-induced NO and prostaglandin E2 production in alveolar macrophages [14]. In addition, ER alleviated reactive oxygen species (ROS) generation and regulated the NF-κB pathway to protect endothelial cells from high glucose-induced apoptosis [15]. This research suggested that ER may be able to inhibit the production of inflammatory cytokines such as TNF-α, IL-1β and IL-6 in vitro or in vivo. Inflammation and oxidative stress are closely related to ulcerative colitis. Therefore, we hypothesized that ER might have a similar colitis-ameliorating effect to rosmarinic acid.

Dextran sulfate sodium (DSS) could destroy the colonic mucosal barrier, leading to the infiltration of granulocytes, the polarization of macrophages and the secretion of inflammatory factors. The DSS-induced UC animal model, due to the humanlike symptoms, simple operation and good repeatability, is extensively used to assess the effect of drugs on UC. And the disease activity index (DAI) score, colonic histopathology and the levels of some inflammatory factors (NO, TNF-α, IL-1β and IL-6) are the necessary indicators to evaluate the progression of UC [18,19,20]. Myeloperoxidase (MPO) activity reflects the degree of neutrophil infiltration and is also a sign of inflammation of UC [21]. Moreover, metabolites in an organism are closely related to physiological and pathological changes [22,23]. Metabolomics has been applied to the diagnosis and treatment of UC patients [24,25]. Therefore, metabolomics analysis is an effective way to explore the effect of the anti-UC drug.

In the present study, the protective effect of ER against UC was evaluated using a lipopolysaccharide (LPS)-induced RAW264.7 cell inflammation model and DSS-induced UC mice model, and the mechanism was explored based on the serum and colon metabolomics studies.

## 2. Results

### 2.1. Effect on LPS-Induced RAW264.7 Cell

#### 2.1.1. Cell Viability

**Figure 1:**
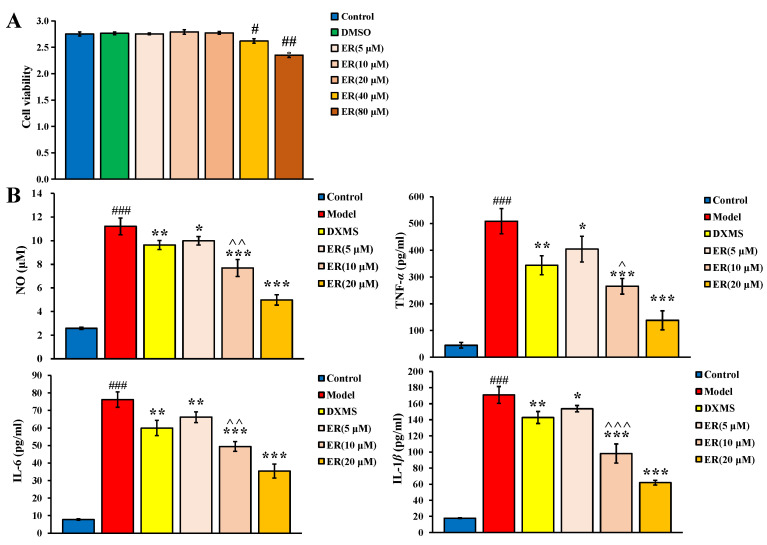
(**A**) Effect of ER on RAW264.7 cell viability. (**B**) Effects of ER on NO, TNF-α, IL-1β and IL-6 levels in LPS induced RAW264.7 cells. (Data were expressed as the means ± standard deviation (SD). Compared with control group, # *p* < 0.05, ## *p* < 0.01, ### *p* < 0.001; compared with model group, * *p* < 0.05, ** *p* < 0.01, *** *p* < 0.001; compared with DXMS group, ^ *p* < 0.05, ^^ *p* < 0.01, ^^^ *p* < 0.001).

The result of the RAW264.7 cell viability assay is shown in Figure 1A. Compared with the control group, low dosages (5 μM, 10 μM and 20 μM) of ER had no significant effect on the survival rate of cells, but 40 μM and 80 μM of ER could significantly inhibit the cell viability (*p* < 0.05, *p* < 0.01). Therefore, 5 μM, 10 μM and 20 μM were chosen as the dosage of ER to investigate the anti-inflammatory activity.

#### 2.1.2. Anti-Inflammatory Activity

As shown in Figure 1B, the levels of NO, IL-6, TNF-α and IL-1β of the model group were all significantly increased compared with the control group (*p* < 0.001), which showed the inflammation was successfully induced. After the intervention of ER or dexamethasone (DXMS), compared with the model group, the levels of NO, TNF-α, IL-1β and IL-6 could be significantly inhibited to various degrees (*p* < 0.05, *p* < 0.01, *p* < 0.001). Notably, the inhibitory effect of ER was even better than DXMS of the same dosage (*p* < 0.05).

The above research results showed that ER had good anti-inflammatory activity in a dose-dependent manner in vitro.

### 2.2. Effect on DSS-Induced Mice

#### 2.2.1. DAI

**Figure 2:**
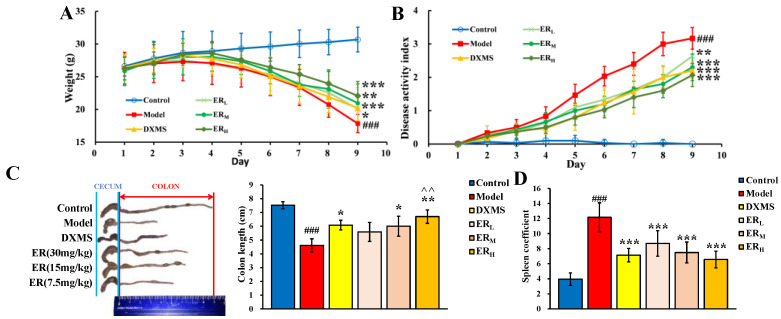
Effect of ER on (**A**) body weight, (**B**) disease activity index, (**C**) colon length and (**D**) spleen coefficient. (Data are expressed as the means ± SD (*n* = 10). Compared with control group, ### *p* < 0.001; compared with model group, * *p* < 0.05, ** *p* < 0.01, *** *p* < 0.001, compared with DXMS group, ^^ *p* < 0.01).

Throughout the experiment, the mice in the control group (Control) had a normal diet and gained weight. In contrast, the mice in the model group (Model) gradually showed appetite loss and weight loss. However, after ER or DXMS intervention, the weight loss was alleviated. On the ninth day, the weights of all administration groups were greater than the model group (*p* < 0.05, *p* < 0.01, *p* < 0.001). The daily weight changes are shown in Figure 2A.

In addition, normal mice had dry stools and no anal bleeding, and the DAI score was basically unchanged. Furthermore, UC model mice showed loose stools and blood in the stool, which indicated that the symptoms of UC model mice became more and more obvious with the extension of DSS induction time. As shown in Figure 2B, the DAI scores of all UC model mice increased with modeling time. From day 1 to day 4 of administration, the DAI scores of UC model mice were all significantly increased compared with Control. However, from the fifth day of administration, the increase rate of DAI score of each administration group was significantly lower than that of Model (*p* < 0.05, *p* < 0.01, *p* < 0.001), which illustrated that ER and DXMS could slow down the increase in DAI scores of UC model mice. On day 9, ER treatment could dose-dependently decrease the DAI score (*p* < 0.01, *p* < 0.001) compared with Model, which was similar to DXMS.

In summary, ER could effectively improve the symptoms in UC model mice.

#### 2.2.2. Colon Length and Spleen Coefficient

Both the typical colon photos and the histogram of each group are shown in Figure 2C. Just as shown in the figure, the colon length of the model group was significantly shorter compared to the control group (*p* < 0.001), which indicated the colon tissue of the UC model mice had been damaged. Oral administration of ER or DXMS could reduce the degree of colon damage. The colon length of the high dosage of ER (ER_H_) group was significantly longer than that of Model (*p* < 0.01), and the colon length of the middle dosage of ER (ER_M_) group or DXMS group was also longer than that of Model (*p* < 0.05). In addition, the length of the colon of the ER_H_ group was also longer than that of the DXMS group (*p* < 0.01), indicating that the degree of colon damage of the ER_H_ group was lower than that of the DXMS group.

The effect of ER on the spleen coefficient is shown in Figure 2D. Compared with Control, the spleen coefficient of Model was significantly increased (*p* < 0.001), meaning that the model mice of UC had an inflammatory reaction. While, after the intervention of DXMS or three dosages of ER, the spleen coefficients all significantly decreased compared with Model (*p* < 0.001). The results showed that ER and the positive drug could attenuate the inflammatory response in UC model mice.

#### 2.2.3. Quantification of Cytokines and MPO Contents

**Figure 3:**
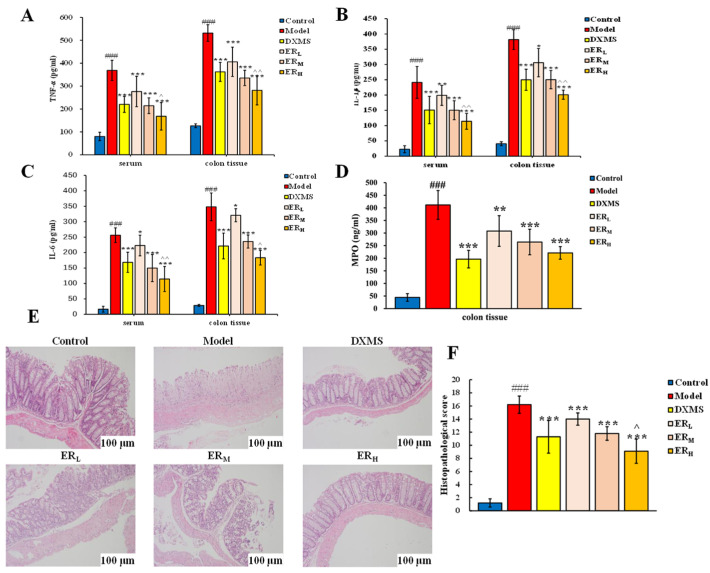
The levels of (**A**) TNF-α, (**B**) IL-1β, (**C**) IL-6 and (**D**) MPO in serum and in colon. (**E**) The typical H&E staining photo and (**F**) histopathological score of the colon section. (Data are expressed as the means ± SD (*n* = 10). Compared with control group, ### *p* < 0.001; compared with model group, * *p* < 0.05, ** *p* < 0.01, *** *p* < 0.001; compared with DXMS group, ^ *p* < 0.05, ^^ *p* < 0.01).

The levels of cytokines and the contents of MPO in serum and in the colon are shown in Figure 3A–D.

In serum, TNF-α, IL-1β and IL-6 in Model all significantly increased compared with Control (*p* < 0.001). After drug intervention, the levels of the above three cytokines all obtained the inhibition. Compared with Model, the levels of the three cytokines in the DXMS, ER_H_ and ER_M_ groups all notably decreased (*p* < 0.001), and TNF-α in the low dosage of ER (ER_L_) group also obviously reduced (*p* < 0.001). Moreover, the increase of IL-1β and IL-6 of the ER_L_ group could also be inhibited compared with Model (*p* < 0.05). It is also worth mentioning that the inhibition effects on three inflammatory cytokines of the ER_H_ group were stronger than the DXMS group (*p* < 0.05, *p* < 0.01).

In the colon, the effects on TNF-α, IL-1β and IL-6 of ER or DXMS were similar to the effects in serum. And ER_H_ showed a stronger effect than DXMS. In addition, the MPO level significantly increased in UC model mice (Figure 3D). After the treatment of DXMS, ER_H_ or ER_M_, the levels of MPO obtained a notable reduction compared with Model (*p* < 0.001). Meanwhile, a low dosage of ER could also decrease the MPO level (*p* < 0.001).

The results of quantitative determination showed that ER could decrease the levels of TNF-α, IL-1β, IL-6 and MPO in a dose-dependent manner in model mice of UC. The effect of ER_H_ was better than DXMS.

#### 2.2.4. Histopathology

The typical H&E staining photos and the histopathological score of the colon section in each group are shown in Figure 3E,F.

Firstly, the H&E staining photos were observed. In the samples from Control, the entire normal colonic structure and mucosal epithelium could be seen. However, severe mucosal damage, edema in the submucosal region, goblet cell depletion, infiltration of inflammatory cells and loss of crypts appeared in the colon tissue of the model group. While the symptoms of colon tissue in the DXMS group and three ER groups were all relieved, including decreasing inflammatory cell infiltration, restoring epithelial damage and relatively intact colon tissue compared with Model.

Secondly, the histopathological scores were evaluated. The average score of Model was about 16, which was greater than the score of Control. With the intervention by ER or DXMS, the histopathological scores were all obviously reduced compared with Model (*p* < 0.001), indicating the symptoms of UC have been significantly improved. In addition, because the score of the ER_H_ group was lower than the DXMS group (*p* < 0.05), it was concluded that the anti-UC effect of ER_H_ was better than DXMS.

#### 2.2.5. Molecular Docking

**Figure 4:**
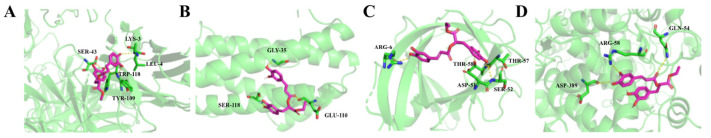
Docking of ER in TNF-α (**A**), IL-6 (**B**), IL-1β (**C**) and MPO (**D**).

The binding energies of ER connected to TNF-α, IL-6, IL-1β and MPO were −28.03 KJ/mol, −24.69 KJ/mol, −27.20 KJ/mol and −24.27 KJ/mol, respectively. The binding mode of ER with each receptor is shown in Figure 4. In the TNF-α binging site, it could be observed that ER was connected to amino acid residues such as SER-43, LYS-3, LEU-4, TRP-110 and TYR-109 through five hydrogen bonds. In the IL-6 binging site, ER was connected to GLY-35, SER-118 and GLU-110 residues through three hydrogen bonds. In the IL-1β binging site, five hydrogen bonds were observed to connect ER with ARG-6, THR-58, THR-57, ASP-51 and SER-52 residues. In the MPO binging site, three hydrogen bonds were observed at ASP-389, ARG-58 and GLN-54 residues connected with ER. The above results illustrated that there was good binding between ER and each receptor.

### 2.3. Metabolomics Study

#### 2.3.1. Validation of UPLC-QTOF-MS

In the system stability test, the *m/z*-retention time (RT, min) pairs chosen in positive electrospray ionization (ESI^+^) mode included 280.0927-0.65; 820.8627-5.09; 184.0744-13.27; 520.3428-17.12; 991.6719-18.11; 184.0756-20.17; 524.3710-20.67; 184.0754-27.13, and the *m/z*-RT pairs in negative electrospray ionization (ESI^−^) mode were as follows: 215.0326-0.65, 818.8585-5.05, 514.2862-8.46, 255.2330-18.07, 492.3458-19.17, 279.2329-23.203, 281.2482-25.04, 283.2635-27.02.

The relative standard deviations (RSDs) of the peak intensities (PI) and RT in system stability, precision, reproducibility and sample stability tests are shown in Table 1.

The above validation indicated that the established UPLC-QTOF-MS method exerted good precision, reproducibility and stability.

**Figure 5:**
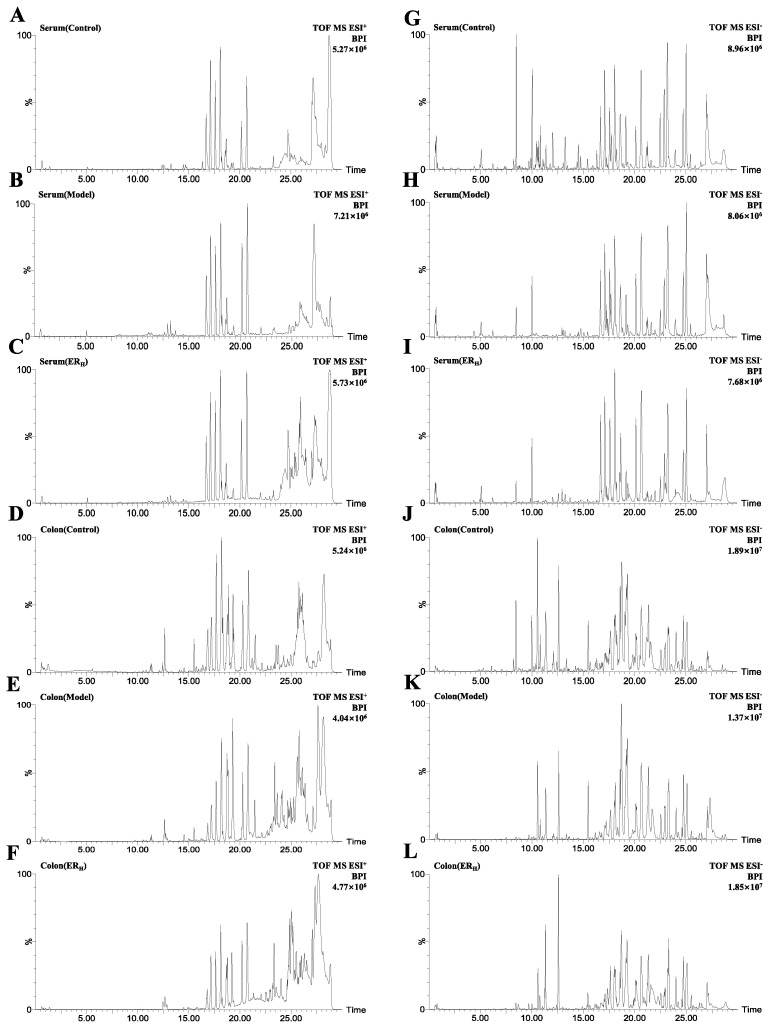
The representative BPI chromatograms of serum and colon samples of Control, Model and ER_H_ groups in (**A**–**F**) positive modes and in (**G**–**L**) negative modes.

Based on the validated method, the serum and colon samples were detected. The representative base peak intensity (BPI) chromatograms of each group are shown in Figure 5.

#### 2.3.2. Multivariate Statistical Analyses of Serum and Colon Metabolomics

**Figure 6:**
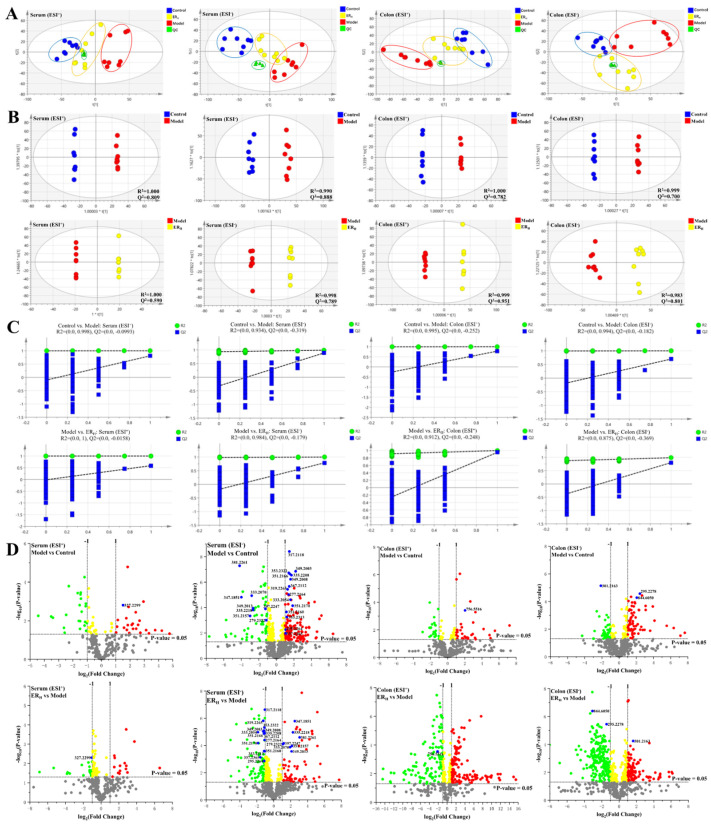
The (**A**) PCA score, (**B**) OPLS-DA score, (**C**) permutations test and (**D**) volcanic plots of serum and colon metabolic profiling.

Principle component analysis (PCA) in ESI^+^ and ESI^−^ were firstly performed, respectively, and the PCA plots are shown in Figure 6A. QC samples were clustered in the central area, indicating that the stability of the system was credible. Different colored dots represented the samples from different groups. The dots of the Control, Model and ER_H_ group, were clearly divided into three different areas, and the dots of the ER_H_ group was located between Control and Model, indicating that the metabolites in the three groups were different, and the metabolites in the ER_H_ group might be re-regulated towards the normal levels.

In order to obtain the maximum separation between two groups, the orthogonal projections to latent structures discriminant analysis (OPLS-DA) model in ESI^+^ and ESI^−^ modes were established, respectively. The satisfactory R^2^ and Q^2^ parameters indicated that the model had good predictive ability and reliability (Figure 6B). Furtherly, permutation tests were performed as shown in Figure 6C, in which the Q^2^-values on the left were lower than the original points on the right, indicating the models were valid. The volcano plots generated to identify the differential metabolites were shown in Figure 6D, and there were 28 differential metabolites identified and marked in blue color. Moreover, red dots represented the up-regulated metabolites (fold change, FC > 2), and green dots represented the down-regulated metabolites (FC < 0.5).

**Figure 7:**
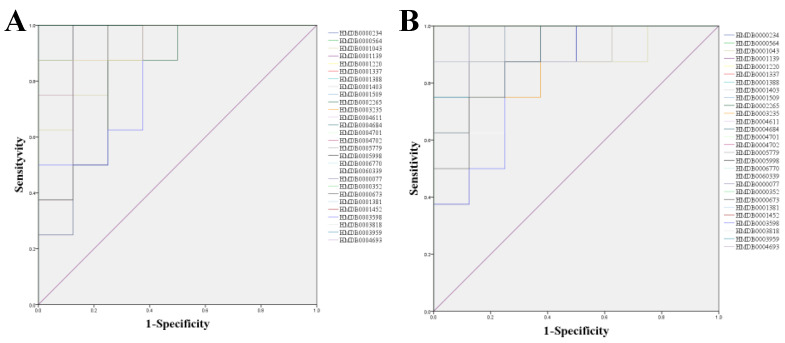
The predictive receiver operating characteristic (ROC) curves (**A**) between Model and Control, (**B**) between Model and ER_H_ group.

Receiver operating characteristic (ROC) curves were then generated with these 28 candidate biomarkers. The area under curve (AUC) values and *p*-values of the 28 biomarkers in two predictive ROC curves are listed in Table 2. The AUC were all greater than 0.8, and *p*-values were all less than 0.05. As shown in the ROC curves for Model and Control (Figure 7A), the 28 differential metabolites could be considered as the potential diagnostic markers for UC disease. The ROC curves for the Model and ER_H_ group (Figure 7B) indicated that the 28 differential metabolites really contributed to the effect of ER on UC.

#### 2.3.3. Biomarkers Screening and Pathway Enrichment

**Figure 8:**
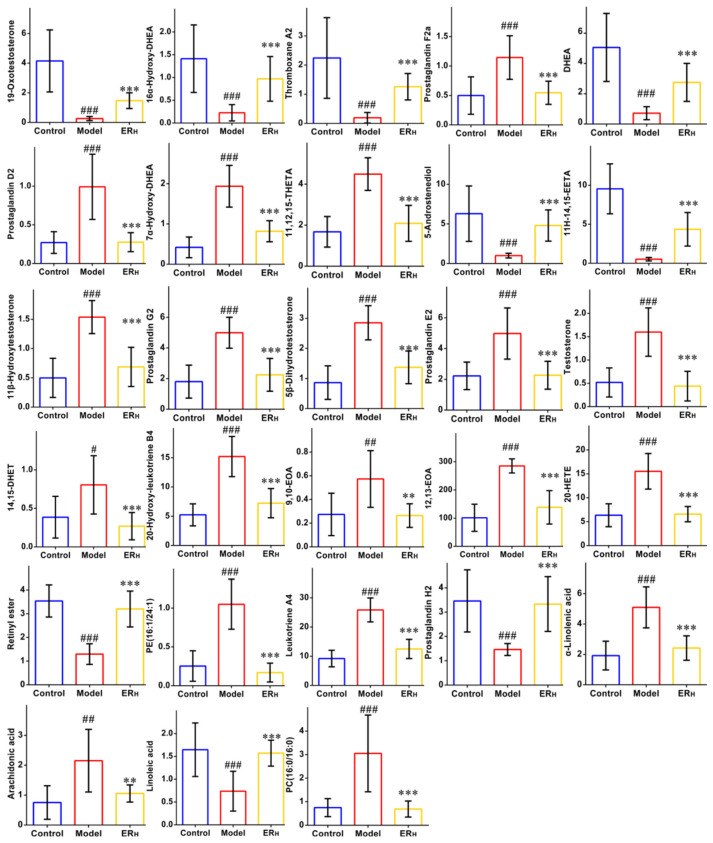
The contents of each biomarker in serum or in colon of Control, Model and ER_H_ group. (Data are expressed as the means ± SD (*n* = 8). Compared with control group, # *p* < 0.05, ## *p* < 0.01, ### *p* < 0.001; compared with model group, ** *p* < 0.01, *** *p* < 0.001).

**Figure 9:**
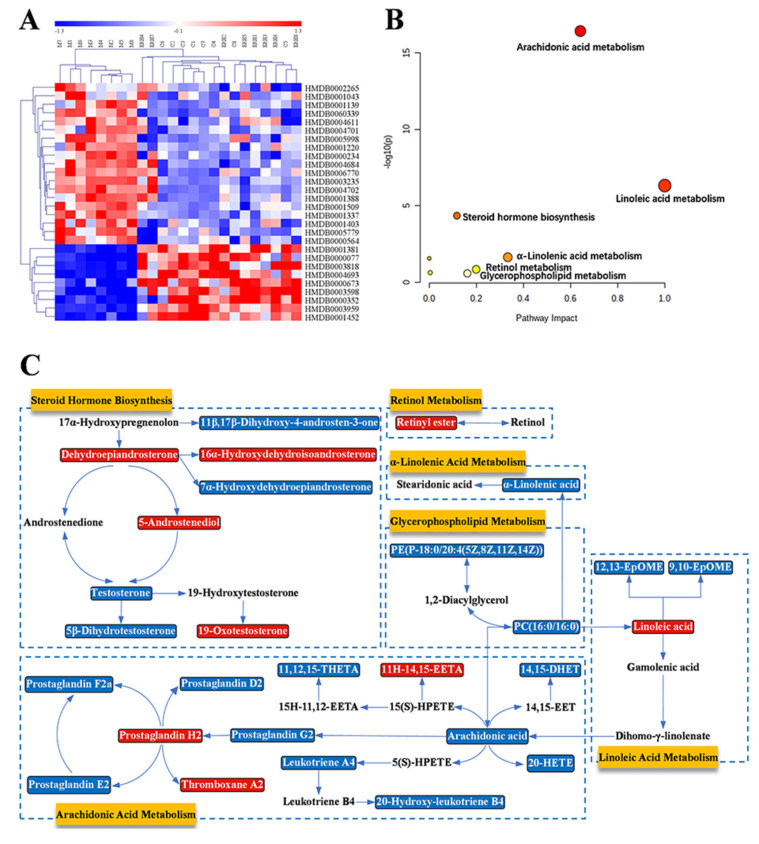
(**A**) The heatmap of biomarkers. (**B**) The metabolisms involved in the therapeutic effects of ER on UC. (**C**) Metabolic network between biomarkers and metabolisms.

The detailed information of the identified 28 biomarkers is listed in Table 3. The content of each biomarker in the serum and colon of the Control, Model and ER_H_ group is shown in Figure 8, respectively. In order to more intuitively simultaneously observe the contents of all biomarkers in different groups, a heatmap was then constructed (Figure 9A). In this heatmap, red color to blue color represented a decreasing abundance of the biomarkers.

The MetaboAnalyst system was then used to perform the pathway enrichment analysis, and the results are shown in Figure 9B and Table 4. The identified 28 biomarkers were found to be involved in six metabolic pathways, of which impacts were greater than 0.1. The six pathways were linoleic acid metabolism (LM), arachidonic acid metabolism (AM), α-linolenic acid metabolism (ALM), glycerophospholipid metabolism (GlyM), retinol metabolism (RM) and steroid hormone biosynthesis (SHB). In addition, the metabolic network based on biomarkers in these six pathways was established to reveal the connection of these biomarkers (Figure 9C). In the network, red metabolites represented the content that was up-regulated, and blue metabolites represented those that were down-regulated with ER treatment which clearly showed that ER_H_ could regulate the disturbance of the six pathways.

The metabolomics study showed that UC disease caused the disturbance of some metabolites and metabolisms, and the disturbance could be re-regulated with the treatment of ER.

## 3. Discussion

UC has been listed by the WHO as one of the refractory diseases in the world [26]. The pathogenesis of UC involves a complex inflammatory response [27,28]. With the gradual increase in the prevalence of UC, natural products with anti-UC effects are receiving more and more attention. For example, coptisine [29], schisandrin B [30] and rosmarinic acid [11].

ER, a natural active ingredient in Labiatae plants such as Salvia chinensis and Perilla frutescens, is increasingly concerned by researchers due to its excellent pharmacological effects. Previous research indicated that ER had anti-inflammation [14], anti-oxidation [15], anti- hypertension [16] and neuroprotective activity [17]. Although ER could be applied to treat some diseases, the role of ER against UC has not been fully reported or elucidated. In the present study, the classical LPS-induced RAW264.7 cells model and DSS-induced mice colitis model were established, which show very similar clinical symptoms to human UC [20]. Our study also further confirmed the important role of inflammatory factors, such as NO, TNF-α, IL-1β and IL-6, in the occurrence and development of ulcerative colitis. Results showed that ER treatment could significantly attenuate UC-related inflammation or other symptoms, including bodyweight loss, fecal blood, diarrhea and colon shortening, and a decreased DAI score in the DSS-induced UC model mice in a dose-dependent manner. In addition, colonic MPO activity is considered to be closely related to the degree of neutrophil infiltration. In our study, the high activity of MPO in UC model mice was notably reduced in the ER-treated groups, which suggested that ER could effectively inhibit neutrophil infiltration.

Histopathology examination, used to examine the pathological changes, is necessary for investigating the disease process that occurs in body organs or tissues. In this study, the colon in UC model mice showed an obvious loss of colonic mucosa epithelium and goblet cells, the apparent absence of crypt structure and the inflammatory cell infiltration. However, the symptoms of colon tissue were significantly relieved or restored by treatment with DXMS or ER.

According to the reports, compared with DXMS, ER showed a stronger ability to inhibit LPS-induced NO and prostaglandin E2 production in alveolar macrophages [14]. A similar situation also appeared in our research. Namely, ER notably exhibited a superior effect towards DXMS both on UC related symptoms and the histopathological examination. Moreover, compared with reported anti-UC natural products (coptisine, schisandrin B or rosmarinic acid), ER has the advantage of a smaller effective dose, which predicts less toxicity or side effects of ER. This indicates that ER may be a novel potential natural agent for patients with UC. In future studies, we could continue to deeply explore the mechanism of ER, or continue to establish other UC animal models (TNBS-induced or Oxazolone-induced UC model) to further confirm the role of ER against UC.

In our metabolomic study, it was elucidated that the 28 biomarkers involved in six metabolisms were obviously interfered with by ER treatment. The six metabolisms were also found in UC patients [31,32,33]. These pathways formed a complex metabolic network. Retinyl ester in retinol metabolism, the storage form of retinol in the body, is a common form of vitamin A, which plays an indispensable role in UC [34]. Except for the retinol metabolism, the other five pathways belong to lipid metabolism pathways, which have been reported to be closely related to the development of inflammation. Among these pathways, the metabolism with the highest impact value was linoleic acid metabolism, which indicated its importance in the development and treatment of UC [22]. Linoleic acid, the synthetic precursor of arachidonic acid, can inhibit the inflammatory response by reducing the yield of inflammation factors such as TNF-α and IL-1 [35]. In addition, there were 14 metabolites, including a variety of prostaglandins, leukotrienes and other inflammatory substances, involved in Arachidonic acid metabolism. These metabolites were found to be re-regulated by the treatment of ER. It was also proven that the intestine was one of the tissues capable of producing and metabolizing steroid hormones. Steroids are fundamental hormones that participate in a wide variety of physiological processes. Most of the metabolites of steroid hormone synthesis identified were related to sex hormones [33]. The risk of UC was increased with sex hormone metabolism disorders. Steroid hormone biosynthesis was found to be disturbed by UC and could be re-regulated with the treatment of ER in our study. Moreover, α-Linolenic acid, a kind of n-3 PUFAs involved inα-Linolenic acid metabolism, played an important role in the treatment of chronic inflammation represented by UC [36]. In glycerophospholipid metabolism, two biomarkers were identified. PC(16:0/16:0) plays a crucial role in lipid metabolism. PE(P-18:0/20:4 (5Z, 8Z, 11Z, 14Z)) is one of the main sources of PC(16:0/16:0). Several key biomarkers, such as arachidonic acid, linoleic acid, α-linolenic acid and PC(16:0/16:0), connected the relevant metabolisms and then formed a metabolic relationship network. To sum up, it is believed that the strong pharmacological effects of ER must be related to its multi-target mechanism.

## 4. Materials and Methods

### 4.1. Chemicals and Reagents

Ethyl rosmarinate, DXMS, linoleic acid, DHEA, α-linolenic acid and arachidonic acid with purity ≥98% were purchased from Sichuan Weikeqi Biological Technology Co., Ltd. (Chengdu, China). 20-HETE with purity ≥98% were purchased from Shanghai Yuanye Bio-Technology Co., Ltd. (Shanghai, China). Prostaglandin F2a, prostaglandin D2, prostaglandin E2, PC(16:0/16:0) and LPS with purity ≥95% were purchased from Merck (Darmstadt, Germany). DSS (MW: 36000–50000 Da) was purchased from MP Biomedicals (Santa Ana, CA, USA).

Cell-grade DMSO was obtained from Solarbio Science & Technology Co., Ltd. (Beijing, China). Roswell Park Memorial Institute 1640 medium (RPMI-1640) and fetal bovine serum (FBS) were obtained from Thermofisher Scientific (Shanghai, China). Penicillin, streptomycin and phosphate buffer saline (PBS) were bought from Servicebio Technology Co., Ltd. (Wuhan, China). High-performance liquid chromatography (HPLC) grade acetonitrile and methanol were purchased from Thermofisher Scientific (Shanghai, China). All other organic solvents were of analytical grade and bought from Anhui Zesheng Technology Co., Ltd. (Hefei, China).

Cell Counting Kit-8 (CCK-8) was bought from Beiren Chemical Technology Co., Ltd. (Beijing, China). ELISA kits specific for mouse MPO, TNF-α, IL-6 and IL-1β were obtained from MultiSciences (Lianke) Biotech, Co., Ltd. (Hangzhou, China). NO kits were purchased from Beyotime Biotechnology Co., Ltd. (Shanghai, China).

### 4.2. Effect on LPS-Induced RAW264.7 Cell

#### 4.2.1. Cell Culture

The RAW264.7 cells were supplied by the Cell Bank of the Chinese Academy of Sciences (Shanghai, China). The cells were incubated at 5% CO_2_ and 37 °C. RPMI-1640 supplemented with 10% FBS and 1% double antibody (penicillin and streptomycin) was chosen as a medium, which was changed once daily. The cells in the logarithmic growth phase were selected for the experiment.

#### 4.2.2. Cell Viability

CCK-8 assay was used to detect cell activity. RAW264.7 cells were seeded (100 μL per well) in 96-well plates at a concentration of 6 × 10^4^ cells/mL and cultured at 5% CO_2_ and 37 °C for 24 h in RPMI-1640 medium containing 10% FBS and 1% double-antibody. Then, the medium was replaced with a new RPMI-1640 medium.

ER was dissolved in DMSO (<2.5‰). The cells were divided into seven groups. Cells in ER groups (5, 10, 20, 40 and 80 μM), the DMSO group and the control group were cultured for 24 h. CCK-8 was then added to the cells, which were incubated for an additional 4 h. The OD value at 450 nm was detected. All the experiments were conducted in six replications.

#### 4.2.3. Anti-Inflammatory Activity

RAW264.7 cells were seeded in 96-well plates at a concentration of 6 × 10^4^ cells/mL (100 μL per well). After culturing 24 h, they were divided into six groups:

1. DMSO control group,
2. Model group;
3. ER 5 μM group;
4. ER 10 μM group;
5. ER 20 μM group;
6. DXMS 10 μM group.

ER and DXMS were dissolved with DMSO (<2.5‰) and were added to the corresponding group for pre-treating for 3 h. Then LPS (1 μg/mL) was added to each group (except the DMSO control group) for 21 h incubation.

The supernatants of each group were then collected for determining the contents of NO, TNF-α, IL-6 and IL-1β using commercial assay kits according to the manufacturer’s instructions.

### 4.3. Effects of ER on DSS-Induced UC in Mice

#### 4.3.1. Experimental Design

BALB/c mice (male, 18–22 g) were obtained from Liaoning Changsheng Biotechnology Co., Ltd. (Shenyang, China, Certificate No. 20210518). All the mice were housed in a standard environment (22–24 °C, relative humidity of 50–60% and 12-h light-dark cycle) and provided with standard food and water for 5 days acclimatization.

Mice were randomly divided into six groups (*n* = 10):

1. Control group (Control);
2. UC-model group (Model);
3. DXMS group (1.0 mg/kg/d);
4. ER_L_ group (7.5 mg/kg/d);
5. ER_M_ group (15 mg/kg/d);
6. ER_H_ group (30 mg/kg/d).

The doses of ER were chosen based on the preliminary toxicity test result, the pre-experiment result and the effective doses of structural analog (rosmarinic acid) [11]. From day 6 to day 13, the mice in groups 2~6 drank DSS aqueous solution (3.5%, *w/v*) ad libitum to induce the UC model, while the mice in 1 group drank the normal water. During this period, the mice in groups 3~6 were intragastrical administered with DXMS or ER once a day, while the mice in Control and Model were administered with distilled water. The volume of administration was all 10 mL/kg. All the mice were weighed and the behavioral status (including fecal characteristics and blood stool) was recorded every day. [22]

On the 14th day, after fasting for 12 h, the mice blood, colon and spleen were taken for analysis.

#### 4.3.2. Disease Activity Index

In order to obtain the quantitative evaluation, the disease activity index (DAI) score was introduced: DAI = (weight loss + fecal characteristics + blood stool)/3. The scores of weight loss, fecal characteristics and blood stool were judged according to Table 5 [37]. The average DAI of each group was calculated every day.

#### 4.3.3. Sample Collection and Preparation

The whole blood was collected by removing the eyeballs, coagulated at 4 °C for 30 min, and centrifuged at 4000× *g* rpm for 10 min to obtain the serum sample for biochemical indicators assay. The serum samples of the Control, Model and ER_H_ group were prepared for metabolomics study.

All mice were sacrificed by cervical dislocation after blood collection. Colons and spleens were then separated. Spleens were weighted and the spleen coefficient was calculated (spleen weight (mg)/body weight (g)). After the colon length was measured, it was dissected along the longitudinal mesentery and rinsed with physiological saline. The colon sample was divided into three parts. The proximal colon was used for biochemical indicators assay. The distal colon was fixed in 4% paraformaldehyde solution and prepared for the subsequent histological evaluation. The middle colon of the Control, Model and ER_H_ group was used for metabolomics study.

#### 4.3.4. Quantification of Cytokines and MPO Contents

The levels of TNF-α, IL-1β and IL-6 in serum were determined using commercial ELISA kits according to the manufacturer’s instructions.

The colon samples were homogenized with pre-cooled PBS to obtain a 10% tissue homogenate, which was centrifuged at 13,000× *g* rpm for 10 min at 4 °C to collect the supernatant. The levels of MPO, TNF-α, IL-1β and IL-6 in the supernatants were finally detected using ELISA kits.

#### 4.3.5. Histopathology

After the colon tissues were fixed in 4% paraformaldehyde solution, they were sequentially paraffin-embedded, sectioned, deparaffinized, hydrated, and H&E stained. The colonic slides were observed under a microscope and photographed. The histological score was evaluated according to the “Histopathology scoring system” [38].

#### 4.3.6. Statistical Analysis

The data were analyzed by SPSS 19.0 software (SPSS Inc., Chicago, IL, USA), and the results were expressed by a mean ± SD. The significant difference was statistically analyzed by t-test. *p* < 0.05 was considered statistically significant.

#### 4.3.7. Molecular Docking

In order to visualize the binding mode of ER on receptors, a molecular docking study was carried out by using Autodock vina (Version 1.0, Scripps Research, San Diego, CA, USA) software. TNF-α, IL-1β, IL-6 and MPO, which had been quantitatively determined in the above activity tests, were chosen as receptors.

Firstly, the molecular structure of ER was downloaded from the PubChem database (https://pubchem.ncbi.nlm.nih.gov/, accessed on 10 October 2021) and saved as an SDF file. The PDB files of TNF-α (5VH4), IL-6 (1ALU), IL-1β (3NJ5) and MPO (6AZP) were downloaded from the Protein Data Bank database (http://www.rcsb.org/pdb, accessed on 10 October 2021). Secondly, the structures of the ER and receptors were preprocessed. Finally, the docking of ER and receptors was performed. The docking results were determined using Pymol software.

### 4.4. Metabolomics Study

#### 4.4.1. Preparation of Serum and Colon Metabolisms Samples

Serum (100 µL) was vortex-mixed with pre-cooled 80% methanol (300 µL) and centrifuged at 10,000× *g* rpm for 10 min at 4 °C. The supernatant was obtained and was freeze-dried. The dried residue, dissolved with 50 µL of 80% methanol, was filtered by a syringe filter (0.22 µm) to obtain serum test solution. In addition, a 5 μL aliquot of each test solution was mixed to acquire the serum QC sample for the method validation.

Colon tissue (0.1 g) was homogenized with 80% methanol (1000 μL), then, according to the above preparation of serum test solution, the colon test solution and the colon QC sample were prepared.

#### 4.4.2. UPLC-QTOF-MS Conditions

Waters ACQUITY UPLC system with Waters Xevo G2-S Q-TOF mass spectrometer was used to perform the sample testing in both positive mode and negative mode.

Conditions for the LC system: ACQUITY UPLC BEH C18 (2.1 mm × 100 mm, 1.7 μm, Waters, Milford, MA., USA) column, column temperature 30 °C, elution procedure: 0.1% formic acid in water (A)-acetonitrile (B) with linear gradient (0.4 mL/min): 10% B (0~2 min); 10→90% B (2~26 min); 90% B (26~28 min); 90→10% B (28~28.1 min); 10% B (28.1~35 min), temperature of sample manager 4 °C, weak wash solvent 10% acetonitrile, strong wash solvent 90% acetonitrile, injection volume 5 μL.

Conditions for the MS system: capillary voltage in positive mode 2.6 kV and in negative mode of 2.2 kV, cone voltage 40 V, source temperature 150 °C, desolvation temperature 400 °C, desolventizing gas flow rate 800 L/h, cone gas flow rate 50 L/h, MSE mode, centroid, low energy 6 V, high energy 20~40 V. QC sample was run randomly four times throughout the whole worklist. Raw data recording was performed on the MassLynx V4.1 workstation (Waters, Manchester, UK).

#### 4.4.3. Validation of UPLC-QTOF-MS

The applied method should be validated in ESI^+^ and ESI^−^ as follow:

- System Stability: A serum QC sample was run randomly to monitor the stability of the system. The exact *m/z*-RT (min) pairs of 16 ions (from different spectral regions) were monitored.
- Precision: The precision was estimated by detecting five consecutive replicates of the serum QC sample in succession.
- Reproducibility: The reproducibility of sample preparation was assessed by analyzing five parallel replicates of one serum sample.
- Sample Stability: The stability of the post-preparation of the sample was evaluated by detecting one serum sample settled in autosampler for 0, 4, 8, 10 and 12 h at 4 °C.

In the above investigation, the RSDs of PI and RT in ESI^+^ and in ESI^−^ should all be calculated.

#### 4.4.4. Analysis Method

The MarkerLynx XS V4.1 software (Waters Co., Milford, MA, USA) was used to perform a series of processing such as calibration, deconvolution, data reduction, chromatographic peak extraction and normalization of the collected data in MarkerLynx software. The identical components in various samples should have the same values of RT and *m/z*. The major processing parameters were set as follows: mass tolerance 0.10, retention time window 0.10, minimum intensity 10%, marker intensity threshold 4000 counts, and noise elimination level 6. Extend Statistics XS viewer could display the *m/z*-RT pairs with intensities for all detected peaks.

The above-acquired data were then multivariate statistically analyzed. In order to separate and classify the samples, PCA was used to evaluate the intrinsic variation of the data lists and to visualize trends between groups. OPLS-DA models were performed to obtain volcano plots and variable importance in the projection (VIP) values. The volcano plots could delineate the statistical significance level and the FC difference of each metabolite. Variables with FC > 2 or <0.5, VIP > 1, *p* < 0.05 were identified as potential biomarkers [39]. The permutation test was carried out to prove the validity of the models in order to promise credible classification results [7]. In addition, the ROC curve was used to validate the markers, in which the AUC should be greater than 0.8 [7]. The above results were determined using Simca 14.1 software (Umetrics, Malmo, Sweden) and Origin 2018 software (OriginLab, Northampton, MA, USA).

Some biochemical databases, including HMDB (https://hmdb.ca/, accessed on 15 October 2021), KEGG (http://www.kegg.com/, accessed on 21 November 2021), METLIN (http://metlin.scripps.edu/, accessed on 21 October 2021) and MetaboAnalyst (https://www.metaboanalyst.ca/, accessed on 21 October 2021) were used to further identify potential biomarkers. Potential biomarkers were eventually confirmed via comparison with reference standards or MS/MS fragments.

MetaboAnalyst (https://www.metaboanalyst.ca/, accessed on 21 October 2021) was finally used to screen the metabolic pathways. The pathways with impact >0.10 were considered as the signal pathways [7].

## 5. Conclusions

In the current study, an LPS-induced RAW264.7 cell inflammation model, DSS-induced UC mouse model and serum and colon metabolomics techniques were used to evaluate the activity of ER in vivo and in vitro for the first time. The results showed that under the intervention of ER, the levels of NO, TNF-α, IL-1β, IL-6 and MPO in the cell supernatant, serum or colon were significantly decreased, and the disease activity index and colon tissue damage were also effectively improved or restored. In addition, 28 biomarkers and six metabolisms were found to be re-regulated by ER in UC model mice. In summary, ER could effectively ameliorate the progression of UC and could be used as a new natural agent for the treatment of UC.

## Figures and Tables

**Table 1:** The RSDs (%) of PI and RT in validation tests.

Tests	ESI^+^ Mode		ESI^−^ Mode	
	PI	RT	PI	RT
system stability	1.35~3.72	0.84~2.36	1.56~3.22	0.24~2.92
precision	0.94~3.28	0.10~0.43	0.61~3.42	0.06~0.34
reproducibility	1.01~3.69	0.12~1.76	0.38~3.74	0.09~0.72
sample stablilty	1.33~3.27	0.08~0.54	1.92~3.41	0.11~0.69

**Table 2:** The AUC values and *p*-values of the biomarkers in ROC curves.

HMDB	Model vs. Control		ER_H_ vs. Model	
	AUC	*p*	AUC	*p*
HMDB0000077	1.000	<0.001	0.969	<0.001
HMDB0000234	1.000	<0.001	1.000	<0.001
HMDB0000352	1.000	<0.001	1.000	<0.001
HMDB0000564	0.984	<0.001	1.000	<0.001
HMDB0000673	0.828	<0.001	0.828	<0.001
HMDB0001043	0.922	<0.01	0.844	<0.01
HMDB0001139	0.906	<0.001	0.984	<0.001
HMDB0001220	0.984	<0.001	0.938	<0.001
HMDB0001337	1.000	<0.001	1.000	<0.001
HMDB0001381	0.984	<0.001	0.922	<0.001
HMDB0001388	1.000	<0.001	0.969	<0.001
HMDB0001403	1.000	<0.001	1.000	<0.001
HMDB0001452	0.969	<0.001	0.953	<0.001
HMDB0001509	1.000	<0.001	0.984	<0.001
HMDB0002265	0.813	<0.05	0.969	<0.001
HMDB0003235	1.000	<0.001	1.000	<0.001
HMDB0003598	0.922	<0.001	0.891	<0.001
HMDB0003818	1.000	<0.001	1.000	<0.001
HMDB0003959	1.000	<0.001	1.000	<0.001
HMDB0004611	1.000	<0.001	0.906	<0.001
HMDB0004684	1.000	<0.001	0.953	<0.001
HMDB0004693	1.000	<0.001	1.000	<0.001
HMDB0004701	0.922	<0.01	0.969	<0.01
HMDB0004702	0.984	<0.001	0.906	<0.001
HMDB0005779	1.000	<0.001	1.000	<0.001
HMDB0005998	0.906	<0.001	0.953	<0.001
HMDB0006770	1.000	<0.001	0.953	<0.001
HMDB0060339	0.859	<0.001	0.875	<0.001

**Table 3:** The information of identified metabolites in serum and in colon.

No.	RT	Compound Name	Mass	Δm	Adducts	Source	HMDB ID	Pathway	Change Trend	
									M/C	ER_H_/M
1 ^a^	8.46	19-Oxotestosterone	347.1851	0.15	M + FA−H	Serum	HMDB0003959	SHB	↓	↑
2 ^a^	9.05	16α-Hydroxy-DHEA	349.2013	0.15	M + FA−H	Serum	HMDB0000352	SHB	↓	↑
3 ^a^	9.81	Thromboxane A2	351.2157	0.00	M−H	Serum	HMDB0001452	AM	↓	↑
4 *	10.33	Prostaglandin F2a	353.2313	0.00	M−H	Serum	HMDB0001139	AM	↑	↓
5 *	11.16	DHEA	333.2070	0.16	M + FA−H	Serum	HMDB0000077	SHB	↓	↑
6 *	11.45	Prostaglandin D2	351.2170	0.00	M−H	Serum	HMDB0001403	AM	↑	↓
7 ^a^	11.51	7α-Hydroxy-DHEA	349.2003	0.15	M + FA−H	Serum	HMDB0004611	SHB	↑	↓
8 ^a^	11.55	11,12,15-THETA	353.2322	0.00	M−H	Serum	HMDB0004684	AM	↑	↓
9 ^a^	12.01	5-Androstenediol	335.2218	0.15	M + FA−H	Serum	HMDB0003818	SHB	↓	↑
10 ^a^	12.01	11H-14,15-EETA	381.2261	0.13	M + FA−H	Serum	HMDB0004693	AM	↓	↑
11 ^a^	12.65	11β-Hydroxytestosterone	349.2008	0.15	M + FA−H	Serum	HMDB0060339	SHB	↑	↓
12 ^a^	12.78	Prostaglandin G2	367.2112	0.00	M−H	Serum	HMDB0003235	AM	↑	↓
13 ^a^	13.67	5β-Dihydrotestosterone	335.2208	0.15	M + FA−H	Serum	HMDB0006770	SHB	↑	↓
14 *	13.71	Prostaglandin E2	351.2160	0.00	M−H	Serum	HMDB0001220	AM	↑	↓
15 ^a^	14.05	Testosterone	333.2054	0.16	M + FA−H	Serum	HMDB0000234	SHB	↑	↓
16 ^a^	14.26	14,15-DHET	337.2365	0.00	M−H	Serum	HMDB0002265	AM	↑	↓
17 ^a^	14.57	20-Hydroxy-leukotriene B4	351.2166	0.00	M−H	Serum	HMDB0001509	AM	↑	↓
18 ^a^	16.92	9,10-EOA	295.2264	0.00	M−H	Serum	HMDB0004701	LM	↑	↓
19 ^a^	17.25	12,13-EOA	295.2278	0.00	M−H	Colon	HMDB0004702	LM	↑	↓
20 *	18.64	20-HETE	319.2265	0.00	M−H	Serum	HMDB0005998	AM	↑	↓
21 ^a^	18.72	Retinyl ester	301.2163	0.00	M−H	Colon	HMDB0003598	RM	↓	↑
22 ^a^	19.12	PE(16:1/24:1)	844.6050	0.06	M + FA−H	Colon	HMDB0008981	GlyM	↑	↓
23 ^a^	19.7	Leukotriene A4	317.2118	0.00	M−H	Serum	HMDB0001337	AM	↑	↓
24 ^a^	20.58	Prostaglandin H2	397.2247	0.13	M + FA−H	Serum	HMDB0001381	AM	↓	↑
25 *	21.35	α-Linolenic acid	277.2164	0.00	M−H	Serum	HMDB0001388	ALM	↑	↓
26 *	22.35	Arachidonic acid	327.2299	0.07	M + Na	Serum	HMDB0001043	AM	↑	↓
27 *	23.97	Linoleic acid	279.2322	0.00	M−H	Serum	HMDB0000673	LM	↓	↑
28 *	25.05	PC(16:0/16:0)	756.5516	0.03	M + Na	Colon	HMDB0000564	AM, LM, ALM, GlyM	↑	↓

RT, Retention Time, min; Mass, Measured mass, Da; Δm, Relative Deviation, ppm; * Metabolites validated with standards; ^a^ Metabolites confirmed by MS/MS fragments; “C” represents control group; “M” represents model group; “↑” represents the content was up-regulated; “↓” represents the content was down-regulated; ”FA”: Formic acid; “DHEA”: Dehydroepiandrosterone; “THETA”: Trihydroxyeicosatrienoic acid; “EETA”: Epoxyeicosatrienoic acid; “DHET”: Dihydroxyeicosatrienoate; “EOA”: Epoxyoctadecenoic acid; “HETE”: Hydroxyeicosatetraenoic acid; “PE”: Phosphatidylethanolamine; “PC”: Phosphatidylcholine.

**Table 4:** Metabolisms involved in the effect of ER on model mice of UC.

No.	Pathway Name	Match Status	*p*	−log(*p*)	Impact
1	Linoleic acid metabolism	4/5	9.44 × 10^−7^	6.0252	1.00
2	Arachidonic acid metabolism	14/36	7.16 × 10^−16^	15.145	0.64
3	α-Linolenic acid metabolism	2/13	3.12 × 10^−2^	1.5053	0.33
4	Glycerophospholipid metabolism	2/36	1.85 × 10^−1^	0.73213	0.20
5	Retinol metabolism	1/16	3.00 × 10^−1^	0.52327	0.16
6	Steroid hormone biosynthesis	8/77	1.59 × 10^−4^	3.7974	0.12

**Table 5:** Disease activity index score.

Score	Weight Loss (%)	Fecal Characteristics	Blood Stool
0	None	Normal	Negative
1	1–5		
2	5–10	Loose stools	Slight bleeding
3	10–15		
4	>15	Diarrhea	Gross bleeding

## Data Availability

The data presented in this study are available on request from the corresponding author.

## Abbreviations

ANOVA	Analysis of variance
AUC	Area under curve
BPI	Base peak intensity
CCK-8	Cell Counting Kit-8
DAI	Disease activity index
DHEA	Dehydroepiandrosterone
DHET	Dihydroxyeicosatrienoate
DMSO	Dimethyl sulfoxide
DSS	Dextran sulfate sodium
DXMS	Dexamethasone
EETA	Epox-yeicosatrienoic acid
ELISA	Enzyme-linked immunosorbent assay
EOA	Epoxyoctadecenoic acid
ER	Ethyl rosmarinate
ER_H_	High dosage of ER
ER_L_	Low dosage of ER
ER_M_	Middle dosage of ER
ESI	Electrospray ionization
FA	Formic acid
FBS	Fetal bovine serum;
FC	Fold change
H&E	Hematoxylin and eosin stain
HETE	Hydroxyeicosatetraenoic acid
IL-1β	Interleukin-1β
IL-6	Interleukin-6
LPS	Lipopolysaccharide
MPO	Myeloperoxidase
NO	Nitric oxide
OPLS-DA	Orthogonal projections to latent structures discriminant analysis
PBS	Phosphate buffer saline
PC	Phosphatidyl-choline
PCA	Principle component analysis
PDB	Protein Data Bank
PE	Phosphatidylethanolamine
PI	Peak intensities
PUFA	Polyunsaturated fatty acids
QC	Quality control
QTOF-MS	Quadrupole time-of-flight mass spectrometry
ROC	Receiver operating characteristic
RPMI-1640	Roswell Park Memorial Institute 1640 medium
RSD	Relative Standard Deviation
RT	Retention Time
SD	Standard deviation
THETA	Trihydroxyeicosatrienoic acid
TNBS	2,4,6-Trinitro-Benzenesulfonic acid
TNF-α	Tumor necrosis factor-α
UC	Ulcerative colitis
UPLC	Ultra-high-performance liquid chromatography
VIP	Variable importance in the projection
